# Sensitive and rapid detection of *Babesia* species in dogs by recombinase polymerase amplification with lateral flow dipstick (RPA-LFD)

**DOI:** 10.1038/s41598-022-25165-7

**Published:** 2022-11-29

**Authors:** Warunya Onchan, Onchira Ritbamrung, Phanupong Changtor, Waranee Pradit, Siriwadee Chomdej, Korakot Nganvongpanit, Puntita Siengdee, Urasri Suyasunanont, Kittisak Buddhachat

**Affiliations:** 1 Department of Biology, Faculty of Science, Naresuan University, Phitsanulok, 65000 Thailand; 2 Department of Biology, Faculty of Science, Chiang Mai University, Chiang Mai, 50200 Thailand; 3 Excellence Center in Veterinary Bioscience, Chiang Mai University, Chiang Mai, 50200 Thailand; 4 Department of Veterinary Biosciences and Public Health, Faculty of Veterinary Medicine, Chiang Mai University, Chiang Mai, 50200 Thailand; 5 Chulabhorn Graduate Institute, Program in Applied Biological Sciences, Chulabhorn Royal Academy, Kamphaeng Phet 6 Road, Laksi, 10210 Bangkok Thailand

**Keywords:** Biotechnology, Molecular biology, Diseases

## Abstract

Canine babesiosis is a tick-borne disease caused by *Babesia* spp., which infects and destroys healthy erythrocytes, leading to mortality and morbidity in dogs. The diagnosis of babesiosis is tedious and time-consuming, especially in latent and chronic infections. Here, a recombinase polymerase amplification combined with a lateral flow dipstick (RPA-LFD) assay was developed for rapid and accurate detection of *Babesia* spp. in canine blood specimens based on the 18S rRNA region. The RPA-LFD assay using rpaBab264 gave specificity to *Babesia* spp. in dogs (*B. vogeli* and *B. gibsoni*) without cross-amplification to other parasites (apicomplexans and non-apicomplexans), with detection limit of at least 22.5 copies/μl (0.1 fg/µl) at 40 °C for at least 10 min. The whole process of DNA amplification by RPA and readout by LFD did not exceed 30 min. To determine the performance of the RPA-LFD assay, a total of 30 clinical samples was examined and compared with conventional PCR (cPCR) and multiplex HRM (mHRM). Eight dogs (26.67%) were detected as positive by RPA-LFD, while seven and six were found positive by cPCR and mHRM, respectively. RPA-LFD and cPCR showed high agreement with *Babesia* spp. detection with kappa > 0.9. We confirmed that the dogs were infected by *B. vogeli* from sequences of positive PCR results. Our findings suggested that RPA-LFD using the rpaBab264 assay offered a rapid, accurate, cost-effective and simple method for *Babesia* spp. detection that is feasibly applicable to be rapid kit at a pet hospital or point-of-care testing.

## Introduction

Canine babesiosis (or piroplasmosis) is a canine vector-borne disease (CVBD) caused by intraerythrocytic protozoa of the genus *Babesia*^1^. The virulent parasites, *Babesia vogeli* and *Babesia gibsoni* (smaller piroplasm) are considered primary causes of canine babesiosis that affects both domestic and stray dogs in Thailand, causing moderate and often clinically inapparent infection^2,3^. *Babesia canis* and *Babesia rossi* are also major agents causing babesiosis. *B. canis* is transmitted by *Dermacentor reticulatus* and confined to Europe, whereas *B. rossi* has *Haemaphysalis elliptica* (and possibly *H. leachi*) as specific vectors and is confined to sub-Saharan Africa. The disease is transmitted primarily through attachment and feeding by infected ticks, particularly brown dog ticks (*Rhipicephalus sanguineus* sensu lato) in tropical and subtropical countries^4,5^. The severity of the infection and clinical symptoms are dependent on the parasite species, the presence of concurrent infections and age and immune status of the host^1,6^. Typical clinical symptoms of canine babesiosis are fever, weakness, weight loss, anemia, icterus, hemoglobinuria, splenomegaly, anorexia and, in some cases, death^1,7^. Clinical manifestations of canine babesiosis are similar to infection by other hemoparasites such as *Haptozoon canis*, *Ehrlichia canis* and *Anaplasma platys*, implicated with *R. sanguineus* as common vectors causing hepatozoonosis, ehrlichiosis and anaplasmosis, respectively^8^. A precise diagnosis to identify the causative agent of infected dogs is an essential step for effective treatment as different infectious agents are treated or eliminated by diverse drug administration. For example, *Babesia* infection is treated by atovaquone or clindamycin, *Hepatozoon* infection requires treatment by imidocarb injection, whereas *Erhlichia canis* and *Anaplasma* infection are treated by antibiotics such as doxycycline.

Diagnosis of babesiosis in veterinarian clinics is routinely based on observation of classic clinical manifestation and a microscopic examination as a direct demonstration of intra-erythrocytic morphology in a stained blood smear^9,10^. This method requires a professional investigator for an accurate diagnosis due to the difficulty of species identification with low parasitemia and co-infection^4,11^. It is difficult to distinguish between *Babesia* and *Plasmodium* (especially *P. falciparum*) parasites. Accurate diagnosis is laborious and time-consuming. Serological technologies include an indirect fluorescent antibody test (IFAT) and enzyme-linked immunosorbent assay (ELISA) as specific and sensitive detection methodologies with greater sensitivity and specificity than conventional methods. However, cross-reaction may occur with other apicomplexan parasites and reduce effectiveness in detecting early-stage infection^1,4,12,13^. Recently, molecular diagnosis has become a widely used method for canine hemoparasite detection. The polymerase chain reaction (PCR) assay is considered the gold standard in laboratory examination^14,15^ but this approach is not suitable for point-of-care (POC) at veterinary clinics due to the requirement of sophisticated instruments with complex operation processes and long detection times.

Currently, several techniques are available for the isothermal amplification of nucleic acids such as loop-mediated isothermal amplification (LAMP), rolling circle amplification (RCA), strand displacement amplification (SDA), helicase-dependent amplification (HDA) and recombinase polymerase amplification (RPA). These have been widely and extensively implemented for pathogen detection of African swine fever virus^16,17^, hepatitis B virus^18^, *Babesia gibsoni*^12^ and *Babesia canis*^19^. RPA is an isothermal amplification method following the underlying mechanism of enzymatic activities, operating at 37 to 42 °C within 5 to 30 min as described by Piepenburg et al.^20^, and commercialized by TwistDx^21^. RPA products can be detected in various ways either in real-time with fluorescent probes, post-amplification by agarose gel electrophoresis or direct visual readout format by lateral flow dipstick (LFD). Direct visual detection of RPA products requires endonuclease IV (nfo), an LF probe labeled at the 5′ end with FAM and a specific reverse primer labeled at the 5′ end with biotin. The amplicons are then detected by the naked eye on LFD, which uses biotin-ligand molecules and anti-FAM gold conjugates. Advantages of this method include visual detection, high sensitivity and simplicity. Recent publications have demonstrated that this assay has been successfully applied to detect various infectious agents^22,23^.

This study established an RPA-LFD assay to accurately and rapidly detect *Babesia* infection from canine blood. The specificity and sensitivity of the assay were evaluated using synthetic plasmids and clinical samples to display the performance of the RPA-LFD assay. The RPA-LFD assay allows veterinarians to rapidly and accurately determine if the unhealthy dog is suffering from babesiosis and does not require advanced expertise. This assay enables implementation for onsite testing at every pet hospital and POC with no requirement of sophisticated instruments, leading to appropriate treatment, prevention and control of canine babesiosis.

## Materials and methods

### DNA samples used in this study

DNA samples of piroplasm protozoa including *B. vogeli* and *B. gibsoni* were obtained from dog blood with positive PCR or microscopic examination and *Babesia bovis* and *Theileria* sp. in DNA form were isolated from cow blood. DNA samples of other apicomplexan species included *Plasmodium vivax*, *Trypanosoma evansi*, and *H. canis*. Moreover, DNA samples of non-apicomplexan species with *R. sanguineus*-borne disease including *E. canis* and *A. platys* were used. Almost DNA samples were provided from obtained from the Animal Molecular Diagnostic Service (AMDS) (Chiang Mai, Thailand) excluding *P. vivax* (as gift from Prof.Dr.Chaturong Putaporntip, Department of Parasitology, Faculty of Medicine, Chulalongkorn University). These DNA samples were used for specificity determination of the established assay. A total of 30 DNA samples obtained from AMDS were leftover DNA from routine testing which they were extracted from the blood of dogs with clinical signs of canine hemoparasites (e.g. fever, lethargy, anorexia and anemia). Source information of the 30 canine blood samples was recorded in Supplemental file 1. The DNA samples extracted from canine blood were kept in collection tubes with EDTA as an anticoagulant at room temperature and then stored at − 20 °C before use. Total genomic DNA of the blood specimens was extracted using an innuPREP DNA Mini-Kit (Analytikjena, Germany) according to the manufacturer’s instructions. The quantity and purity of extracted DNA were determined by measuring A_260_ and the ratio of A_260_/A_280_ on a Nano Spectrophotometer (MicroDigital, Korea). All extracted DNA samples were stored at − 20 °C until analyzed. No ethical approval was required for this study because the DNA samples were provided by AMDS, and this was confirmed by the Animal Ethics Committee, Center of Animal Research, Naresuan University (NUCAR) (License number NU-AEE620503).

The synthetic plasmid harboring a partial sequence of the 18S rRNA region of *B. vogeli* and *H. canis* and 16S rRNA sequences of *E. canis* and *A. platys* (retrieved from GenBank, NCBI as accession numbers: MH143394, MK091088, KY594915 and KJ659044, respectively) were synthesized and used to clone plasmid pUC57 vector by Macrogen (Korea). The synthetic plasmid pUC57 harboring either partial 18S or 16S rRNA was used as DNA template for the optimized RPA-LFD assay and analytical sensitivity determination.

### Primer and probe design

**Figure 1:**
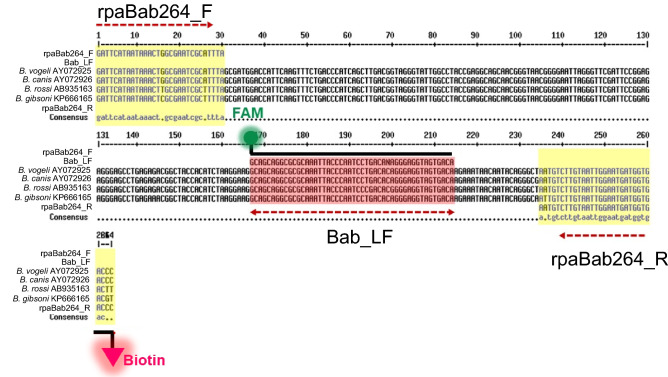
DNA binding sites of rpaBab264 and Bab_LF for *Babesia* spp. detection by nfo RPA assay. Partial 18S rRNA sequences from different species of *Babesia* retrieved from GenBank were aligned by MultAlin.

Partial sequences of the 18S rRNA region of *Babesia* spp. including *Babesia vogeli*, *Babesia canis*, *Babesia rossi*, *Babesia gibsoni* and *Babesia annae*, as well as common canine hemoparasites in Thailand (*H. canis*, *Wuchereria bancrofti*, *Plasmodium falciparum* and *Plasmodium vivax*) and also *Canis lupus familiaris* available in GenBank, NCBI including accession numbers AY072925, AY072926, AB935163, KP666165, KT580785, AY150067.2, AY843438, HQ283221, JQ627158 and AAEX03025866, respectively were performed for multiple alignment by MultAlin (http://multalin.toulouse.inra.fr/multalin/) to determine primer sets for the RPA reaction (Supplemental Fig. 1). The primers and probes were designed following the guidelines of the RPA manufacturer (TwistDx, Cambridge, UK). RPA primer length was between 30 and 35 base pairs, with GC content between 30 and 60% and the reverse primer was labeled at the 5′ end with biotin. The Bab_LF Probe consisted of an antigenic label FAM group at the 5′ end, an internal basic nucleotide analog tetrahydrofuran (THF) spacer and a 3′-polymerase extension blocking group (C3-spacer). The primers and probes were purchased from Eurogentec (Kaneka Corporation, Belgium) and the sequences are shown in Table 1 and Fig. 1.

**Table 1:** Primer and probe sequences used in this study.

Primer/probe name	Oligonucleotide (5’–3’)	%GC content	Tm (°C)	Amplicon size (bp)
rpaBab264	F: GAT TCA TAA TAA ACT GGC GAA TCG CAT TTA	33.3	56.2	264
	R: Biotin-GGG TCA CCA TCA TTC CAA TTA CAA GAC ATT	40	59	
rpaBab128	F; GTA GGG CTA ATA CAC GTT TGA GGT CTT TTG	43.3	59	128
	R: Biotin-ATG GGT CAG AAA CTT GAA TGG TCC ATC GCT	46.7	43.1	
Bab_LF	FAM- GCA GCA GGC GCG CAA ATT ACC CAA TCC TGA CA-THF-AGG GAG GTA GTG ACA -C3-spacer	55.2	71.4	

*F* forward, *R* reverse, *LF* lateral flow, *FAM* carboxyfluorescein, *THF* tetrahydrofuran spacer, *C3 spacer* polymerase extension blocking group.

### Primer screening for RPA

Conventional PCR (cPCR) was carried out to choose suitable primer sets between rpaBab264 and rpaBab128 for *Babesia* spp. detection. PCR amplification was carried out using DreamTaq DNA Polymerase in a total volume of 25 μl containing 12.5 μl of 2X DreamTaq Master Mix (Thermo Fisher Scientific, USA), 0.5 μl of 10 μM forward, 0.5 μl of 10 μM reverse primers (rpaBab264 or rpaBab128), 1 μl of DNA templates (synthetic plasmid of *B. vogeli* at ~ 10^4^ copy number) and 10.5 μl of nuclease-free water. The PCR reaction was performed in a MiniAmp Plus Thermal Cycler (Applied Biosystems, USA) with the following conditions: pre-denaturation at 95 °C for 5 min and 95 °C for 30 s, 55 °C for 30 s, and 72 °C for 45 s for 35 cycles, with a final extension step at 72 °C for 10 min. For RPA amplification, a TwistAmp Liquid Basic Kit (TwistDx, Cambridge, UK) was used. The 25 μl reaction solution contained 12.5 μl of 2X reaction buffer, 0.4 μl of 10 mM dNTP, 2.5 μl of 10X Basic E-mix, 1.2 μl of 10 μM forward primers, 1.2 μl of 10 μM reverse primers, 1.25 μl of 20X Core Reaction mix, 3.7 μl of nuclease-free water and 1 μl of DNA template. To initiate the reaction, 1.25 μl of 280 mM magnesium acetate was added into the tube and then vortexed to mix the solution. The tubes were immediately incubated in a heating block at 40 °C for 20 min. Subsequently, the RPA products were cleaned using NucleoSpin Gel and PCR Clean-up (MACHEREY–NAGEL, USA). Thereafter, the PCR and purified RPA products were subjected to EtBr-containing 1.8% agarose gel electrophoresis (AGE) to visualize the amplicon size under a UV transilluminator.

### Development of RPA-LFD assay

The readout results of *Babesia* spp. detection based on lateral flow dipstick (LFD), the RPA assay were performed using TwistAmp nfo Kit (TwistDx, Cambridge, UK) following the manufacturer’s instructions. Briefly, each reaction contained 29.5 μl of primer-free rehydration buffer, 2.1 μl of 10 μM forward primers (rpaBab264_F) and reverse primers (rpaBab264_R), 0.6 μl of 10 μM Bab_LF Probe, 12.2 μl of nuclease-free water and 1 μl of DNA template. The reaction pellet was resuspended and the reaction was initiated by adding 2.5 μl of 280 mM magnesium acetate. The tubes were then immediately incubated in a heating block at 40 °C for 20 min. As recommended by the manufacturer, the samples were vortexed and spun down after 4 min at first incubation. In each run, a positive control supplied with the TwistAmp nfo Kit (primers/probe and template) and negative control (nuclease-free water) were included. After incubation, the RPA products were purified using NucleoSpin Gel and PCR Clean-up (MACHEREY–NAGEL, USA) and analyzed by agarose gel electrophoresis. The unpurified RPA products were directly detected by lateral flow strips—HybriDetect (Milenia Biotec, Germany) or PCRD nucleic acid detector assay (Abingdon Health, UK). A 5 μl aliquot of RPA product was transferred to a new microcentrifuge tube containing 100 μl of HybriDetect assay buffer and then the LFD strip was then dipped into the mixture for 5 min to visualize the result at room temperature. Next, a 6 μl aliquot of RPA product was pipetted to a new microcentrifuge tube containing 84 μl of the PCRD extraction buffer, followed by adding 75 μl of the diluted reaction mixture to the sample well of a PCRD test cassette. The RPA reaction result was detected by the naked eye from the test line (T line) for HybriDetect or line 2 (FAM/Biotin-labeled amplicon) for PCRD and the control line (C line) on the strips.

DNA harboring a partial 18S rRNA sequence of *B. vogeli* at 2.25 × 10^4^ copies was used as a DNA template to determine the suitable temperature and incubation for RPA-LFD of a synthetic plasmid. Optimal amplification temperature was determined by performing the RPA assay at 25, 30, 35, 40, 45 and 50 °C for 20 min. Optimal incubation time was investigated by varying the time for DNA amplification at 5, 10, 20, 30 and 40 min. The amplification products were analyzed with LFD and AGE following the procedures described above.

### Determination of specificity

DNA samples of different parasite species including piroplasm protozoa (*B. vogeli*, *B. gibsoni*, *B. bovis* and *Theileria* sp.), other apicomplexans (*H. canis*, *P. vivax* and *T. evansi*) and other parasites implicated with brown tick-borne disease (*E. canis* and *A. platys*) were employed to test the specificity of the RPA-LFD assay using rpaBab264 to diagnose *Babesia* spp. Moreover, to assess the inference of other parasites’ DNA to the RPA assay using rpaBab264 for detecting *Babesia* spp, all other parasites’ DNA with or without either *B. vogeli* or *B. gibsoni* was pooled by adding 1 μl of each species’ DNA into a tube as DNA template to assay. RPA assay using rpaBab264 was performed using TwistAmp nfo Kit (TwistDx, Cambridge, UK) as mentioned above. RPA products were pipetted at 6 μl to detect the amplification results using the PCRD nucleic acid detector assay. RPA products were also subjected to agarose gel electrophoresis.

### Determination of analytical sensitivity

Ten-fold serial dilutions of *B. vogeli*-pUC57 plasmid, ranging from 2.25 × 10^6^ (10 pg/µl) to 2.25 × 10^0^ copies/µl, were used to evaluate the sensitivity of RPA-LFD, RPA-AGE and cPCR using rpaBab264 to detect *Babesia* spp. The RPA products obtained from TwistAmp nfo Kit were visualized for the results using HybriDetect. Copy numbers of plasmid molecules were calculated using the equation:

$${\text{number of copies = }} \frac{{{\text{amount}} \left( {{\text{ng}}} \right) \times {6}{\text{.022 }} \times {10}^{{{23}}} }}{{{\text{length}} \left( {{\text{bp}}} \right) \times { 1} \times {10}^{{9}} \times { 660}}}$$

### Validation of RPA-LFD assay by clinical samples

To determine the accuracy of the RPA-LFD assay using rpaBab264 to diagnose *Babesia* spp., a total of 30 DNA samples isolated from clinical blood samples were randomly selected from dogs with clinical signs of canine hemoparasites (e.g. fever, lethargy, anorexia and anemia) from AMDS. The approaches used for *Babesia* detection of specimens with unknown CVBD status were validated using multiplex high resolution melting analysis (mHRM) and cPCR. For mHRM, a reaction of 25-μl volume using SensiFAST HRM Kit (Bioline, UK) followed the protocol of Buddhachat et al.^24^. Both RPA-LFD and cPCR assays for *Babesia* spp. detection followed the procedures described earlier. For readout of the RPA-LFD assay results of clinical samples, RPA products were detected by PCRD nucleic acid detection assay (Abingdon Health, UK). To confirm the result of the RPA-LFD assay, positive PCR products were sequenced at Macrogen (Korea).

### Statistical analysis

Degrees of agreement between the RPA-LFD-cPCR assay and RPA-LFD-mHRM were measured using kappa (K) values, with K < 0.4 considered to be a poor agreement and K ≥ 0.75 considered to be a good agreement. The chi-squared test was used to compare assays at the 0.01 level of significance.

### Ethical approval

The authors confirm that all methods were performed in accordance with the relevant guidelines and regulations.

## Results

### Screening the suitable primer candidates for RPA amplification

**Figure 2:**
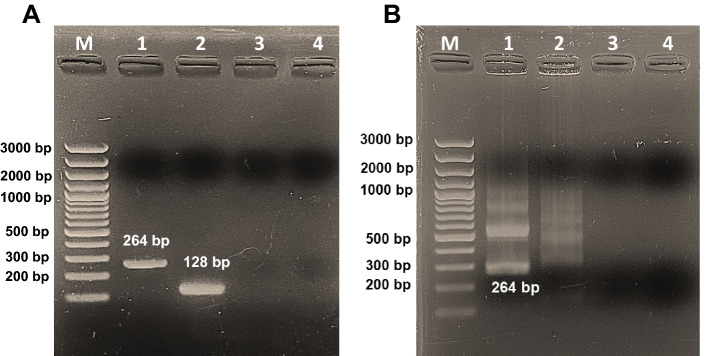
Screening the designed RPA primers by cPCR and RPA. Agarose gel electrophoresis of PCR (**A**) and RPA (**B**) products were generated using designed primers/probes. Lane M: molecular marker; Lanes 1 and 2 are designed primer sets: rpaBab264 and rpaBab128 primers, respectively; Lanes 3 and 4 are negative control (nuclease-free water) for rpaBab264 and rpaBab128, respectively.

Candidate primers/probes targeting the 18S rRNA sequence region were designed and screened for detection of *Babesia* spp. based on the RPA primer design manual. Alignments of the 18S rRNA sequence indicated two regions for the most efficient primer pairs. These regions, conserved between different species of *Babesia*, are generally used for cPCR assays. The rpaBab264 and rpaBab128 primers were designed to amplify high level sequence conservation at the standard plasmid pUC57, with primer length 30 base pairs and GC content accounting for 33.3–46.7%. cPCR and RPA were used to determine the ability of primer pair candidates to amplify DNA. As shown in Fig. 2, specific fragments of 268 and 128 bps were obtained on cPCR-AGE from rpaBab264 and rpaBab128 primers, respectively; however, the rpaBab128 primer was not consistent with RPA amplification using TwistAmp Liquid Basic Kit (the most brilliant band of 128 bp) (Fig. 2A). RPA using rpaBab264 gave the expected size at 264 bp with some non-specific bands (Fig. 2B). Therefore, the rpaBab264 primer was used to perform the RPA-LFD assay for *Babesia* spp. detection.

### RPA condition optimization

**Figure 3:**
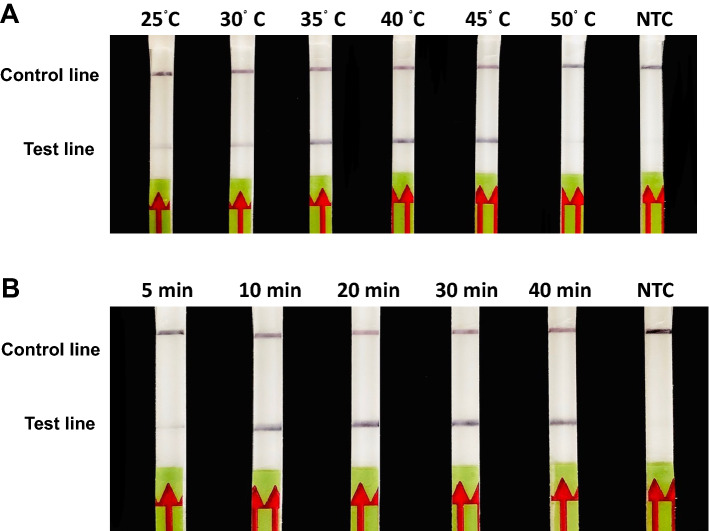
Optimization of the RPA amplification reaction temperature and time. (**A**) Evaluation of different reaction temperatures from 25 to 50 °C. (**B**) Evaluation of different reaction times between 5 and 40 min. Non-template control (NTC) represents the reaction without DNA templates using distilled water.

Optimal conditions for highly efficient RPA amplification were determined at different temperatures and times. Temperature ranges from 25 to 45 °C yielded the band on the test line. The intensity of the band increased up to 40 °C and faded at 45 °C (Fig. 3A). The optimal reaction temperature at 40 °C was selected for downstream assay. For RPA assay incubation time, the 5-min incubation yielded the faded band and then showed the clear band for only 10-min incubation. The intensity of the band appeared constant after 20-min incubation (Fig. 3B). Therefore, the RPA reaction to amplify target DNA was performed at 45 °C for 20 min.

### The specificity determination of RPA-LFD assay

**Figure 4:**
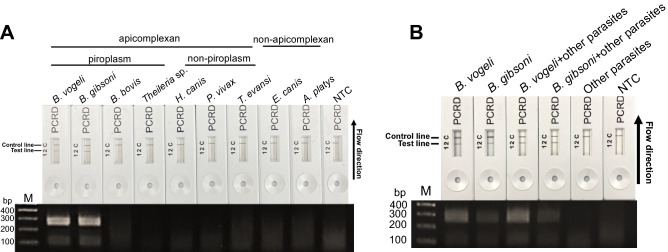
Specificity of the RPA-LFD assay using rpaBab264 for *Babesia* spp. detection and DNA samples of piroplasms, other apicomplexans and non-apicomplexans. The performance of RPA-LFD assay using rpaBab264 was detected by single species per a reaction (**A**) and multiple species per a reaction (**B**). NTC represents the negative control (nuclease-free water).

To determine the specificity of RPA-LFD using rpaBab264, *B. vogeli* and other piroplasms (*B. vogeli*, *B. gibsoni*, *B. bovis* and *Theileria* spp.), other apicomplexans (*H. canis*, *P. vivax* and *T. evansii*,) and non-apicomplexans (*E. canis* and *A. platys*) were used as DNA templates. Results showed the 264 bp amplicon for *B. vogeli* and *B. gibsoni* in RPA-AGE. RPA-LFD results were similar to RPA-AGE with intense bands for *B. vogeli* and *B. gibsoni* and *B. bovis* with a faded band (Fig. 4A). Besides, RPA assay using rpaBab264 appeared not to inference DNA amplification by other parasites’s DNA for detecting both *B. vogeli* and *B. gibsoni* noted by the expected amplicon of both yielded even in the pooled DNA of several parasites (Fig. 4B).

### Sensitivity determination of RPA-LFD assay

**Figure 5:**
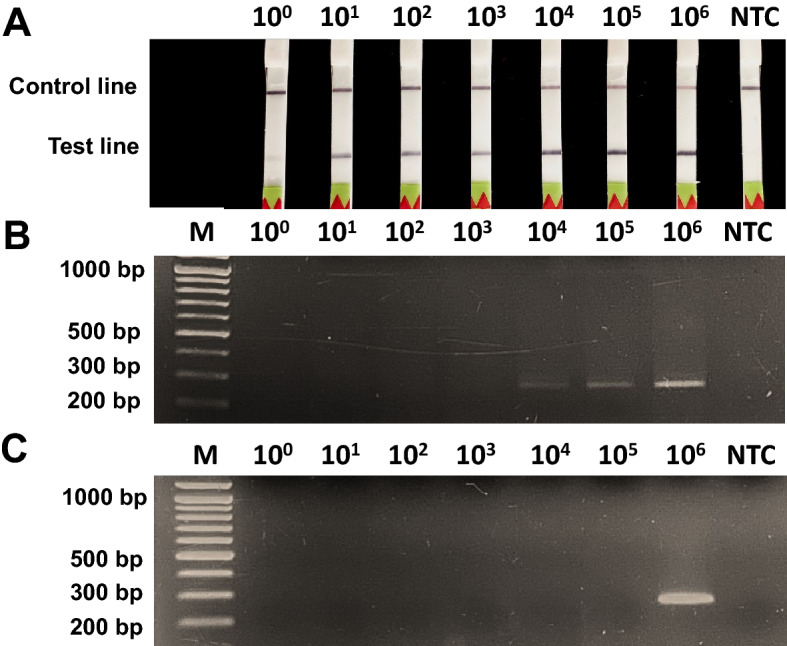
Analytical sensitivity of *Babesia* spp. detection among different approaches including (**A**) RPA-LFD using rpaBab264, (**B**) RPA-AGE and (**C**) cPCR using rpaBab264-AGE. Lane M is DNA ladder, The concentration of DNA used for sensitivity determination ranged from 2.25 × 10^0^ to 2.25 × 10^6^ copies/µl, NTC represents negative control (nuclease-free water).

The sensitivity limit of RPA-LFD was 2.25 × 10^1^ copies/µl (~ 0.1 fg/µl) as visualized by the presence of the control and test lines in lateral flow strips (Fig. 5A), while a clear band at 2.25 × 10^4^ copies/µl was given for RPA-AGE (Fig. 5B). This indicated that the LFD-based detection approach had higher sensitivity than AGE to detect RPA products because double-labeled RPA products (biotin and FAM) were bound on LFD using immunoassays. Sensitivity was also higher than PCR-AGE detection, which was sensitive at only 2.25 × 10^6^ copies/µl (Fig. 5C).

### Validation of RPA-LFD assays by clinical samples

**Figure 6:**
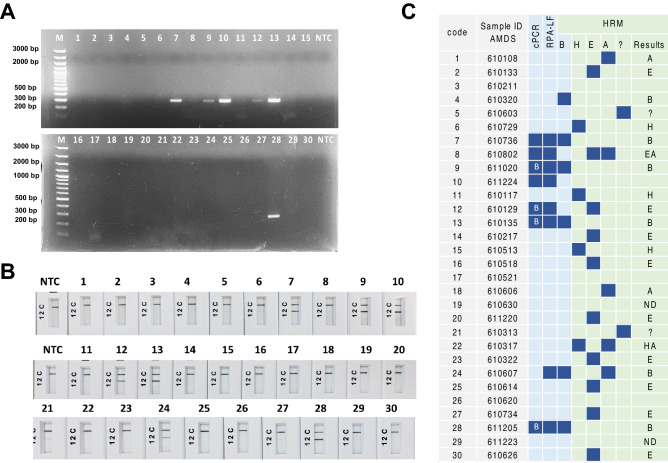
Performance of RPA-LFD using rpaBab264 assay on clinical samples from canine blood compared to cPCR and mHRM. Results were obtained from cPCR (**A**) and RPA-LFD (**B**). Non-template control is abbreviated as NTC. Diagnostic results of three approaches including cPCR, RPA-LFD and mHRM are represented (**C**). mHRM results of 30 clinical samples are illustrated in Supplemental files 2 and 4. Letter “B” in the cPCR results represents sequence results of PCR products matching *B. vogeli*. The symbol “?” represents an unknown peak from mHRM, resulting in an unidentified species. Not detectable (ND) refers to samples with no amplification signal from mHRM. Sequences of codes 9, 12, 13 and 28 were deposited in GenBank as accession numbers OP602997–OP603000, respectively.

The performance of the RPA-LFD assay was evaluated using rpaBab264 to detect *Babesia* spp. DNA samples were isolated from 30 canine blood specimens with clinical manifestations and assessed in parallel with the cPCR and mHRM assays. Our results demonstrated that 23.33% (7/30), 26.67% (8/30) and 20% (6/30) of the samples displayed positive *B. vogeli* for cPCR, RPA-LFD and mHRM (Table 2 and Fig. 6A–C). For mHRM results, the dogs were also infected with *H. canis*, *E. canis*, *A. platys* and an unknown species, with *E. canis* showing the highest infection at 33.33% (10/30) (Fig. 6C and Supplemental file 1 and 2). A few samples (code 5 and 21) showing unknown peaks were recognized as unidentified species (Supplemental files 1 and 3). RPA-LFD and cPCR results exhibited a high degree of agreement at more than 85% in contrast to RPA-LFD and mHRM (Table 2). Results were consistent with the chi-squared test, demonstrating that RPA-LFD—cPCR and RPA-LFD—mHRM assays gave similar performances for *Babesia* spp. detection. However, when performing the kappa statistic test, RPA-LFD and cPCR (K = 0.91) showed a higher degree of agreement when compared to RPA-LFD and mHRM (K = 0.63) (Table 2). Four positive PCR products including 9, 12, 13 and 28 were successfully sequenced, out of seven, revealing similarity to *B. vogeil* with 99–100% identity. These sequences were deposited in GenBank as OP602997- OP603000 (Supplemental file 3).

**Table 2:** Comparative performances of RPA-LFD to cPCR and RPA-LFD to mHRM assays for *Babesia* spp. detection.

Assay		RPA-LFD			Chi-squared	Kappa coefficient
		Positive	Negative	Total		
cPCR	Positive	7	0	7	6.10 × 10^–6^	0.91 (0.74–1.00)
	Negative	1	22	23		
	Total	8	22	30		
mHRM	Positive	5	1	6	0.00276	0.63 (0.30–0.96)
	Negative	3	21	24		
	Total	8	22	30

## Discussion

Babesiosis is one of the major tick-borne dog diseases caused by *Babesia* spp. including *B. rossi*, *B. vogeli*, *B. canis*, *B. gibsoni* (small piroplasms) and *B. microti*-like (*Theileria annae*), transmitted by different tick vectors such as *Haemaphysalis elliptica*, *Dermacentor* spp. and *R. sanguineus*, which are specific to a species of *Babesia*^25^. In Thailand, *B. vogeli* is predominantly persistently present with prevalence ranging from 1.4 to 19.5%^26–29^. Globally, various species are responsible for canine babesiosis as mentioned above. The treatment of canine babesiosis often is oral atovaquone or clindamycin for *Babesia* infection. Here, we developed a specific and rapid diagnostic method for *Babesia* spp. to detect intracellular erythrocytic protozoa from the blood of dogs using recombinase amplification polymerase with lateral flow dipstick (RPA-LFD) as a facile, fast, sensitive and accurate method. This method resolved the low sensitivity limitations of microscopic observation and the requirement of expensive instruments such as PCR or real-time PCR commonly absent in pet hospitals. Our findings showed that the RPA-LFD assay using the 18S rRNA sequence region enabled diagnosis of *Babesia* spp. (*B. vogeli* and *B. gibsoni*) from canine blood without cross-reaction with other hemoparasites such as *B. bovis*, *Theileria* sp. *H. canis*, *P. vivax*, *T. evansi*, *E. canis* and *A. platys*, accompanied by high sensitivity with detection limit of 2.25 copies/µl and 10^5^-fold more sensitive than cPCR.

In this study, the primers were designed to be specific to the various species of *Babesia* that infect dogs. We retrieved the 18S rRNA sequences of these agents from GenBank due to high copy numbers in dog genomes^30^. Consequently, two primer sets were designed, given as rpaBab264 and rpaBab128 for performing cPCR and RPA. For cPCR, both primer sets yielded DNA products that matched the expected size, whereas RPA using rpaBab128 did not work due to the absence of the 128 bp amplicon. Using isothermal DNA amplification methods to detect pathogens provide rapidity and sensitivity but lacks specificity compared to cPCR since non-specific or unexpected amplification can occur because of the lack of the high-temperature denaturation step^31^. Thus, at least two primer sets for RPA should be designed for screening.

The working volume of RPA (25-μl per reaction) used in this study was minimized as half of the recommended volume from the TwistAmp Kit instructions (50 μl per reaction), resulting in similar performance. Several previous studies showed the effect of RPA volume reaction on RPA performance and small volumes of RPA at more than 10 μl did not affect the RPA product^32,33^. Difference in the reaction volume of RPA-LFD using rpaBab264 between 50 μl and 25 μl was also compared, revealing no difference in RPA product (Supplemental Fig. 2). The minimized reaction volume of RPA enabled reduction of cost for pathogen detection and can be adopted for other applications of readout such as microfluid platforms.

Evaluation of *Babesia* spp. detection by RPA-LFD using rpaBab264 showed the ability to amplify *B. vogeli* and *B. gibsoni* with a clear band and no cross-reaction of *B. bovis*, *Theileria* sp. *H. canis*, *P. vivax*, *T. evansi*, *E. canis* and *A. platys*, indicating the specificity of the assay to *Babesia* having a specific vector as *R. sanguineus*. This result concurred with previous studies on the optimization of RPA-LFD assays^32,34,35^. This method also showed no cross-reaction to *R. sanguineus*-borne diseases such as hepatozoonosis (*H. canis*), ehrlichiosis (*E. canis*) and anaplasmosis (*A. platys*). This is unsurprising because both *E. canis* and *A. platys* are recognized as rickettsiae bacteria with no 18S rRNA sequence regions. The genome of *H. canis* protozoa contains the 18S rRNA sequence region and the primer binding site and probe of rpaBab264 have variations resulting in DNA amplification of *H. canis*. The detection limit of RPA-LFD for *Babesia* spp. was assayed at 0.1 fg/µl and was 10^5^-fold more sensitive than RPA visualized by agarose gel electrophoresis. The RPA-LFD assay was more sensitive because the signal on LFD was enhanced by gold nanoparticles on anti-FAM. Cui et al.^12^ established RPA-LF using cytochrome oxidase subunit I (*coxI*) for specific *B. gibsoni*, which is a major epidemiology in China with high specificity and sensitivity. *B. gibsoni* is an infectious agent responsible for canine babesiosis and various species of the *Babesia* genus including *B. rossi*, *B. vogeli*, *B. canis*, *B. gibsoni* and *B. microti*-like cause babesiosis. Here, we developed RPA-LFD as a broad-spectrum for *Babesia* spp. detection to enable wide-ranging diagnosis of *Babesia*-infected dogs and facilitate implementation of the approach in diverse regions occupied by different *Babesia* species. Recently, the timely diagnosis of *B. gibsoni* by a QubeMDx PCR system was achieved with sensitivity limits between 0.000576 and 0.002% of parasitemia. The entire process was completed within 30 min, demonstrating a novel and reliable point-of-care testing method in clinical stations^36^.

The established RPA-LFD assay using rpaBab264 was validated to diagnose the presence of *Babesia* spp. from 30 DNA samples isolated from dogs with clinical manifestations, and all DNA samples were detected for *Babesia* spp. by cPCR and mHRM. RPA-LFD using rpaBab264 and cPCR using rpaBab264 (K = 0.91) gave stronger agreement than RPA-LFD and mHRM (K = 0.63) because the primer sets used in mHRM differed from other the two methods. mHRM used two primer sets for protozoa and rickettsia species that enabled simultaneous detection for four species (*Babesia* spp., *H. canis*, *E. canis* and *A. platys*) at one reaction^24^. Although many samples were negative for *Babesia* spp., mHRM results demonstrated that most samples were infected with *H. canis*, *E. canis* or *A. platys* and even co-infected. However, a few samples had unknown peaks obtained from mHRM (melting temperature at ~ 81.0–81.5 °C) which did not match the known peaks of four species and these were given as unidentified species^24^. Their amplified amplicons require to be sequenced to confirm species identity in further study. We confirmed the positive PCR results by sequencing and four samples exhibited sequence similarity to *B. vogeli* with 99–100% identity. Although mHRM offers superior ability to perform multiple species detection, it requires a real-time PCR machine; thus, this method is unfeasible in pet hospitals for POC testing due to high cost and the need of specialized personnel^24^.

RPA-LFD has shown many strengths in pathogen detection but still requires many improvements to augment effectiveness and versatility and become practically employed at point-of-care. RPA-LFD enables diagnosis of the presence or absence of specific pathogens but it is not a quantitative method. Sensitivity of the RPA-LFD assay was investigated. The intensity of the band on LFD was dose-dependent and it was difficult to see the actual amount of agent infected. The band on RPA-LFD was detected at the end-point product and RPA rapidly saturated even at low concentration of DNA target. It was not correlated to the number of agents. TwistAmp exo enables a quantitative method that requires fluorescence detection^37^, contributing to unfeasibility for point-of-care testing. Moreover, rapid DNA isolation is an essential step to reduce constraints in field implementation and promote RPA-LFD as a practical point-of-care assay. This study performed DNA extraction using a kit that took one hour and required centrifugation that consumed energy. RPA has been reported to enable high tolerance from many inhibitors in various tissues (saliva, urine and blood)^38^. Rapid DNA isolation such as tris–EDTA buffer-based extraction, methanol extraction, sodium hydroxide-based extraction, Chelex DNA extraction and paper-based extraction^39,40^ should be investigated for further study to select a compatible rapid DNA extract to RPA-LFD using rpaBab264 and reduce assay time. This will be more feasible for on-site-testing or in pet hospitals by technicians or veterinarians who are non-experts in microscopic examination.

RPA-LFD provides an effective and feasible point-of-care approach to detect *Babesia* spp. Providing a simple and effective DNA extract from blood to shorten processing time is a vital step for practical use in pet clinics. HUDSON (Heating Unextracted Diagnostic Samples to Obliterate Nucleases) is a simple nucleic acid extraction for CRISPR-Cas-based pathogen detection. DNA obtained from HUDSON can be amplified by RPA and CRISPR-cas13^38^. Multiplex detection for different hemoparasite species by CRISPR-cas based on nucleic acid detection should be further investigated because clinal manifestations are similar for babesiosis, hepatozoonosis, ehrlichiosis and anaplasmosis and can cause co-infection. Timely and accurate diagnosis is vital for disease treatment and management.

## Conclusions

RPA-LFD using the rpaBab264 assay for *Babesia* spp. detection offers a simple, rapid, reliable and cost-effective tool for veterinary clinical stations. This method requires minimal training and uses simple instruments such as a water bath, heat box and incubator. Further optimization of this RPA-LFD technique should focus on developing simple DNA extraction methods that can generate a point-of-care test that is easy to apply in the field. RPA-LFD Kits are expensive and produced by a single manufacturer. Reducing the cost of the reagents will also help to increase availability of the RPA assay.

## Supplementary Information

Supplementary Information 1.

Supplementary Information 2.

Supplementary Information 3.

Supplementary Information 4.

Supplementary Information 5.

Supplementary Information 6.

## Data Availability

All datasets are available in the GenBank, NCBI repository as MH143394, MK091088, KY594915 and KJ659044 for generating plasmid used as positive DNA template for assay, and AY072925, AY072926, AB935163, KP666165, KT580785, AY150067.2, AY843438, HQ283221, JQ627158 and AAEX03025866 for primer design. Sequences of positive PCR product codes 9, 12, 13 and 28 were deposited in the GenBank, NCBI repository as accession numbers OP602997- OP603000, respectively.

## Abbreviations

AGE: Agarose gel electrophoresis
CVBD: Canine vector-borne disease
ELISA: Enzyme-linked immunosorbent assay
FAM: 6-Carboxyfluorescein
HAD: Helicase-dependent amplification
IFAT: Indirect fluorescent antibody test
LAMP: Loop-mediated isothermal amplification
LFD: Lateral flow dipstick
PCR: Polymerase chain reaction
RCA: Rolling cycle amplification
RPA: Recombinase polymerase amplification
SDA: Strand displacement amplification
THF: Tetrahydrofuran

## References

[1] Irwin PJ (2009). Canine babesiosis: From molecular taxonomy to control. Parasit. Vectors.

[2] Kaewkong W, Intapan PM, Sanpool O, Janwan P (2014). High throughput pyrosequencing technology for molecular differential detection of *Babesia vogeli*, *Hepatozoon canis*, *Ehrlichia canis* and *Anaplasma platys* in canine blood samples. Ticks Tick Borne Dis..

[3] Piratae S, Sukumolanan P (2017). Molecular detection of blood pathogens and their impacts on levels of packed cell volume in stray dogs from Thailand. Asian Pac. J. Trop. Dis..

[4] Solano-Gallego L, Sainz Á, Roura X, Estrada-Peña A, MiróA G (2016). A review of canine babesiosis: the European perspective. Parasit. Vect..

[5] Do T, Phoosangwalthong P, Kamyingkird K, Kengradomkij C, Chimnoi W, Inpankaew T (2021). Molecular detection of tick-borne pathogens in stray dogs and *Rhipicephalus sanguineus* sensu lato ticks from Bangkok Thailand. Pathogens.

[6] Li XW, Zhang XL, Huang HL, Li WJ, Wang SJ, Huang SJ, Shao JW (2020). Prevalence and molecular characterization of *Babesia* in pet dogs in Shenzhen, China. Comp. Immunol. Microbiol. Infect. Dis..

[7] Welzl C, Leisewitz AL, Jacobson LS, Vaughan-Scott T, Myburgh E (2001). Systemic inflammatory response syndrome and multiple-organ damage/dysfunction in complicated canine babesiosis. J. S. Afr. Vet. Assoc..

[8] Solano-Gallego L, Trotta M, Carli E, Carcy B, Caldin M, Furlanello T (2008). *Babesia canis* and *Babesia canis vogeli* clinicopathological findings and DNA detection by means of PCR-RFLP in blood from Italian dogs suspected of tick-borne disease. Vet. Parasitol..

[9] Ionita M, Mitrea IL, Pfister K, Hamel D, Buzatu CM, Silaghi C (2012). Canine babesiosis in Romania due to *Babesia canis* and *Babesia vogeli*: a molecular approach. Parasitol..

[10] Zygner W, Gójska O, Rapacka G, Jaros D, Wedrychowicz H (2007). Hematological changes during the course of canine babesiosis caused by large *Babesia* in domestic dogs in Warsaw (Poland). Vet. Parasitol..

[11] Schoeman JP (2009). Canine babesiosis. J. Vet. Res..

[12] Cui J, Zhao Y, Sun Y, Yu L, Liu Q, Zhan X, Li M, He L, Zhao J (2018). Detection of *Babesia gibsoni* in dogs by combining recombinase polymerase amplification (RPA) with lateral flow (LF) dipstick. Parasitol. Res..

[13] Birkenheuer AJ, Levy MG, Stebbins M, Poore M, Breitschwerdt E (2003). Serosurvey of antiBabesia antibodies in stray dogs and American pit bull terriers and American Staffordshire Terriers from North Carolina. J. Am. Anim. Hosp. Assoc..

[14] Jefferies R, Ryan UM, Muhlnickel CJ, Irwin PJ (2003). Two species of canine *Babesia* in Australia: Detection and characterization by PCR. J. Parasitol..

[15] Rucksaken R, Maneeruttanarungroj C, Maswanna T, Sussadee M, Kanbutra P (2019). Comparison of conventional polymerase chain reaction and routine blood smear for the detection of *Babesia canis*, *Hepatozoon canis*, *Ehrlichia canis*, and *Anaplasma platys* in Buriram Province, Thailand. Vet. World.

[16] Dokphut A, Boonpornprasert P, Songkasupa T, Tangdee S (2020). Development of a loop-mediated isothermal amplification assay for rapid detection of African swine fever. Vet. Integr. Sci..

[17] Wang J, Liu J, Yang J, Liu Z, Wang X, Li Y (2019). Molecular detection and genetic diversity of *Babesia canis* in pet dogs in Henan Province, China. Parasitol. Int..

[18] Yao C, Xiang Y, Deng K, Xia H, Fu W (2013). Sensitive and specific HBV genomic DNA detection using RCA-based QCM biosensor. Sens. Actuators B: Chem..

[19] Wang Y, Dai J, Liu Y, Yang J, Hou Q, Ou Y, Ding Y, Ma B, Chen H, Li M, Sun Y, Zheng H, Zhang K, Wubshet AK, Zaberezhny AD, Aliper TI, Tarasiuk K, Pejsak Z, Liu Z, Zhang Y, Zhang J (2021). Development of a potential penside colorimetric LAMP assay using neutral red for detection of African swine fever virus. Front. Microbiol..

[20] Piepenburg O, Williams CH, Stemple DL, Armes NA (2006). DNA detection using recombination proteins. PLoS Biol..

[21] Lobato IM, O'Sullivan CK (2018). Recombinase polymerase amplification: basics, applications and recent advances. Trends Analyt. Chem..

[22] Daher RK, Stewart G, Boissinot M, Bergeron MG (2016). Review recombinase polymerase amplification for diagnostic applications. Clin. Chem..

[23] Castellanos-gonzalez A, Melby P, Travi B (2018). Molecular diagnosis of protozoan parasites by recombinase polymerase amplification. Acta Trop..

[24] Buddhachat K, Meerod T, Pradit W, Siengdee P, Chomdej S, Nganvongpanit K (2020). Simultaneous differential detection of canine blood parasites : Multiplex high-resolution melting analysis (mHRM). Ticks Tick Borne Dis..

[25] Solano-Gallego L, Baneth G (2011). Babesiosis in dogs and cats-expanding parasitological and clinical spectra. Vet. Parasitol..

[26] Buddhachat K, Meesong O, Nganvongpanit K, Osathanunkul M, Chomdej S (2012). Molecular characterization and detection of *Babesia canis vogeli* in asymptomatic roaming dogs in Chiang Mai, Thailand. Thai J. Vet. Med..

[27] Simking P, Wongnakphet S, Stich RW, Jittapalapong S (2010). Detection of *Babesia vogeli* in stray cats of metropolitan Bangkok, Thailand. Vet. Parasitol..

[28] Piratae S, Pimpjong K, Vaisusuk K, Chatan W (2015). Molecular detection of *Ehrlichia canis*, *Hepatozoon canis* and *Babesia canis vogeli* in stray dogs in Mahasarakham province, Thailand. Ann. Parasitol..

[29] Laummaunwai P, Sriraj P, Aukkanimart R, Boonmars T, Wonkchalee N, Boonjaraspinyo S, Sangmaneedet S, Mityodwong T, Potchimplee P, Khianman P, Maleewong W (2014). Molecular detection and treatment of tick-borne pathogens in domestic dogs in Khon Kaen, Northeastern Thailand. Southeast Asian J. Trop. Med. Public Health..

[30] Oyamada M, Davoust B, Boni M, Dereure J, Bucheton B, Hammad A, Itamoto K, Okuda M, Inokuma H (2005). Detection of *Babesia canis rossi*, *B. canis vogeli*, and *Hepatozoon canis* in dogs in a village of Eastern Sudan by using a screening PCR and sequencing methodologies. Clin. Vaccine Immunol..

[31] Burcu Ö, Stephanie EM (2021). A review of reaction enhancement strategies for isothermal nucleic acid amplification reactions. Sens. Actuators B: Chem..

[32] Hu J, Huang R, Sun Y, Wei X, Wang Y, Jiang C, Geng Y, Sun X, Jing J, Gao H, Wang Z, Dong C (2019). Sensitive and rapid visual detection of *Salmonella* Typhimurium in milk based on recombinase polymerase amplification with lateral flow dipsticks. J. Microbiol. Methods..

[33] Zhou S, Zheng X, Yang Z, Huang Q, Yi J, Su L, Guo B, Xiu Y (2022). Development of two recombinase polymerase amplification EXO (RPA-EXO) and lateral flow dipstick (RPA-LFD) techniques for the rapid visual detection of *Aeromonas salmonicida*. Mar. Biotechnol..

[34] Gumaa MM, Cao X, Li Z, Lou Z, Zhang N, Zhang Z, Zhou J, Fu B (2019). Establishment of a recombinase polymerase amplification (RPA) assay for the detection of *Brucella* spp. infection. Mol. Cell. Probes..

[35] Hou P, Zhao G, Wang H, He C, Huan Y, He H (2018). Development of a recombinase polymerase amplification combined with lateral-flow dipstick assay for detection of bovine ephemeral fever virus. Mol. Cell. Probes.

[36] Liu IL, Chi NY, Chang CL, Hung ML, Chiu CT, Chen HW (2019). A novel PCR-based point-of-care method enables rapid, sensitive and reliable diagnosis of *Babesia gibsoni* infection in dogs. BMC Vet. Res..

[37] Gao F, Jiang JZ, Wang JY, Wei HY (2018). Real-time isothermal detection of Abalone herpes-like virus and red-spotted grouper nervous necrosis virus using recombinase polymerase amplification. J. Virol. Methods.

[38] Myhrvold C (2018). Field-deployable viral diagnostics using CRISPR-Cas13. Science.

[39] Bereczky S, Martensson A, Gil JP, Farnert A (2005). Rapid DNA extraction from archive blood spots on filter paper for genotyping of *Plasmodium falciparum*. Am. J. Trop. Med. Hyg..

[40] Gan W, Zhuang B, Zhang P, Han J, Li CX, Liu P (2014). A filter paper-based microdevice for low-cost, rapid, and automated DNA extraction and amplification from diverse sample types. Lab Chip.

