# Supplementary material for: Sensitive and rapid detection of Babesia species in dogs by recombinase polymerase amplification with lateral flow dipstick (RPA-LFD)
Source: Sci Rep. 2022 Nov 29;12:20560. doi: 10.1038/s41598-022-25165-7 (PMC9707278; doi:10.1038/s41598-022-25165-7)
Supplement: Supplementary file 1 — Supplementary Information 1. [file 41598_2022_25165_MOESM1_ESM.pptx]

## Slide 1
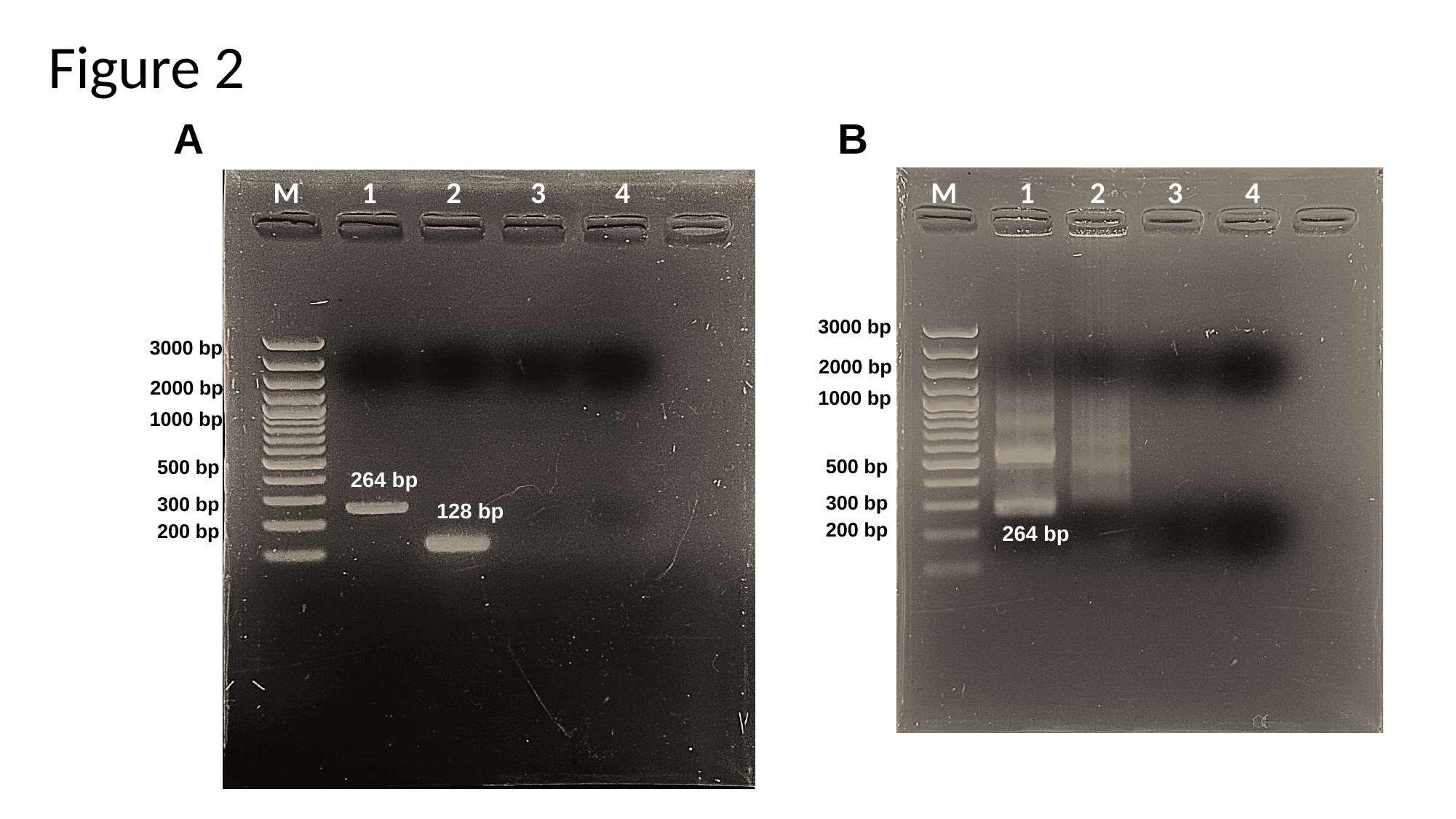

Figure 2
A
B
M 1 2 3 4
M 1 2 3 4
3000 bp
3000 bp
2000 bp
2000 bp
1000 bp
1000 bp
500 bp
500 bp
264 bp
300 bp
300 bp
128 bp
200 bp
200 bp
264 bp

## Slide 2
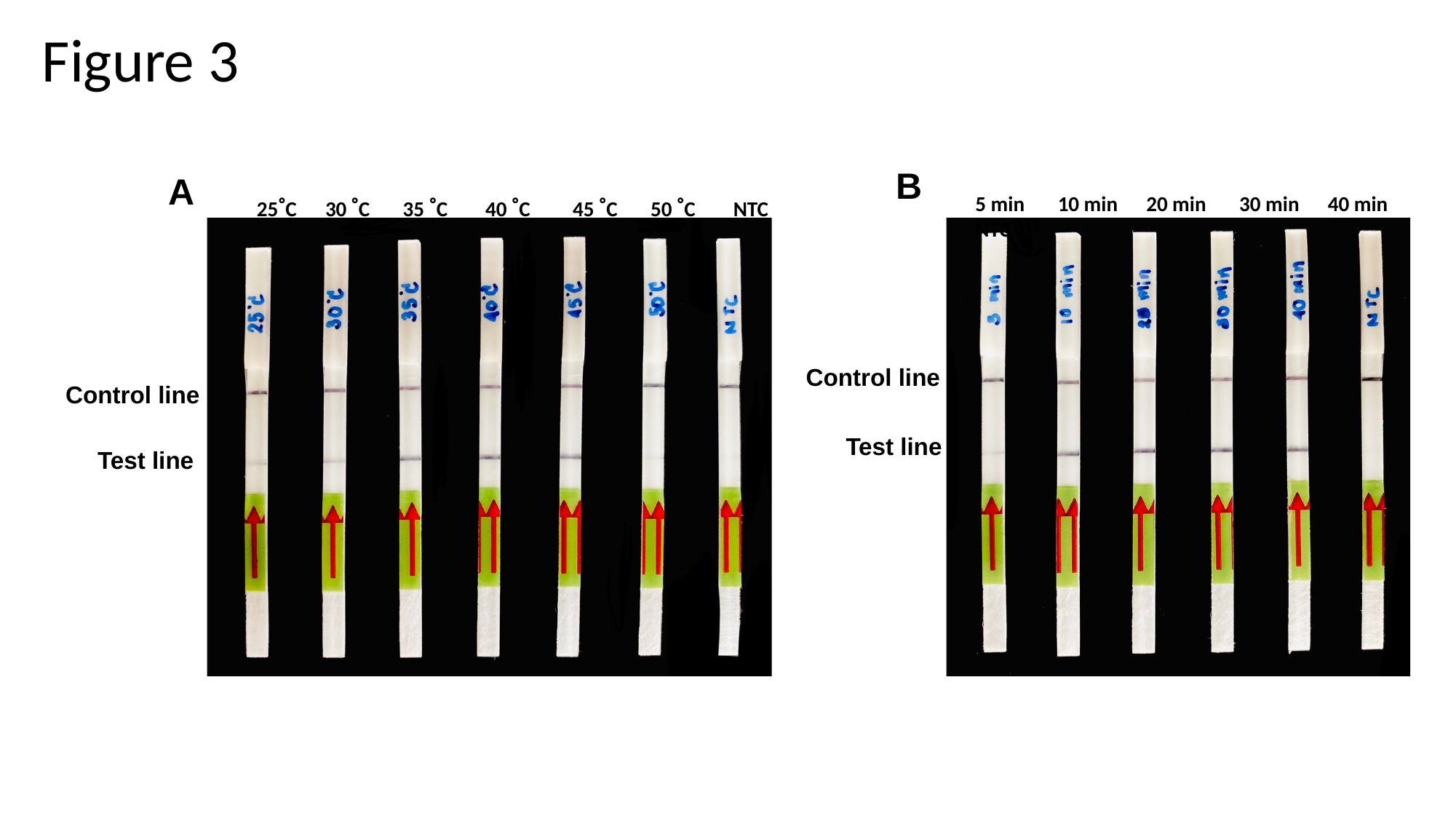

Figure 3
B
A
5 min 10 min 20 min 30 min 40 min NTC
25ﹾC 30 ﹾC 35 ﹾC 40 ﹾC 45 ﹾC 50 ﹾC NTC
Control line
Control line
Test line
Test line

## Slide 3
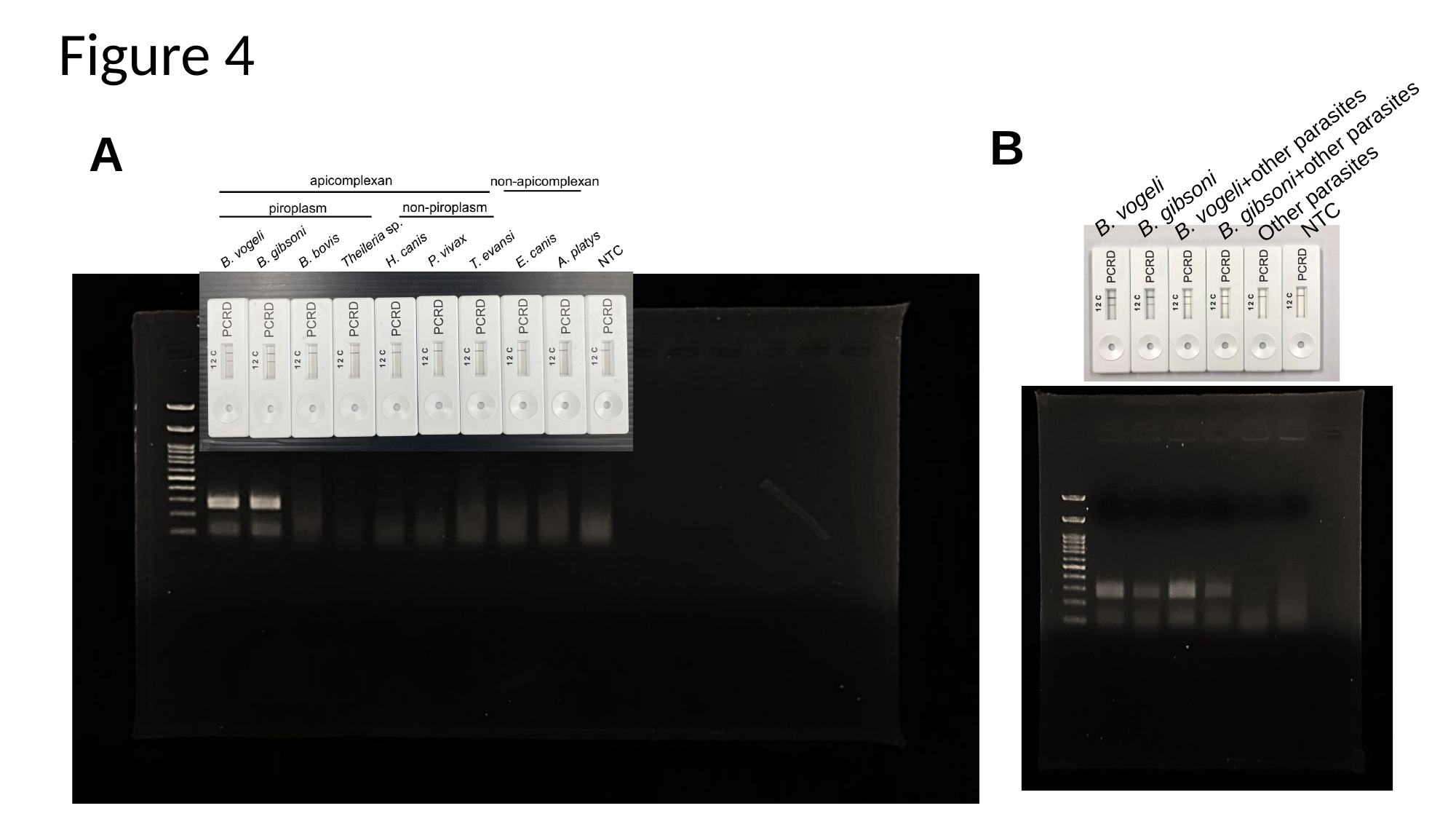

Figure 4
B. gibsoni+other parasites
B. vogeli+other parasites
B
A
Other parasites
B. vogeli
NTC
B. gibsoni

## Slide 4
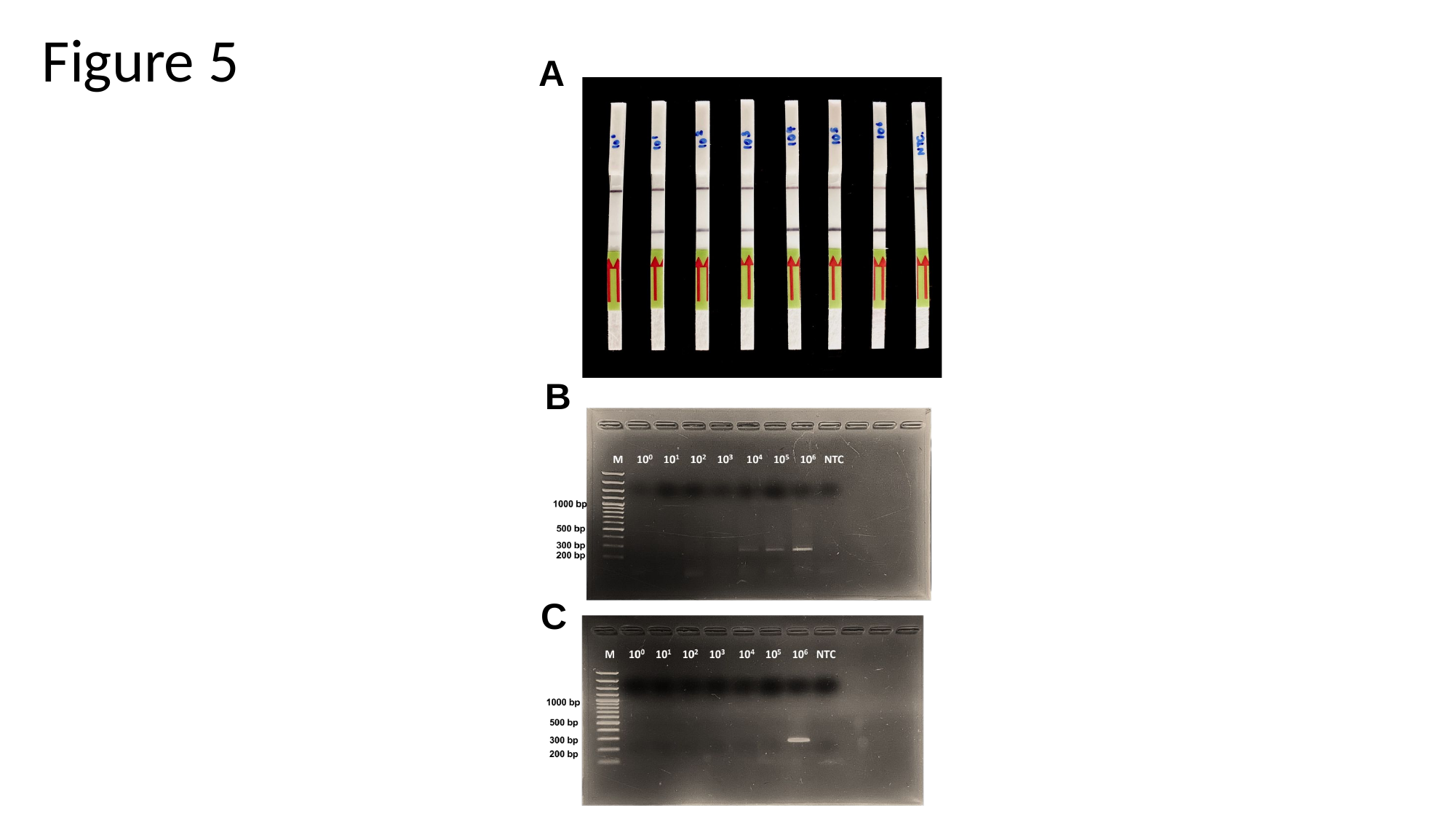

Figure 5
A
B
C

## Slide 5
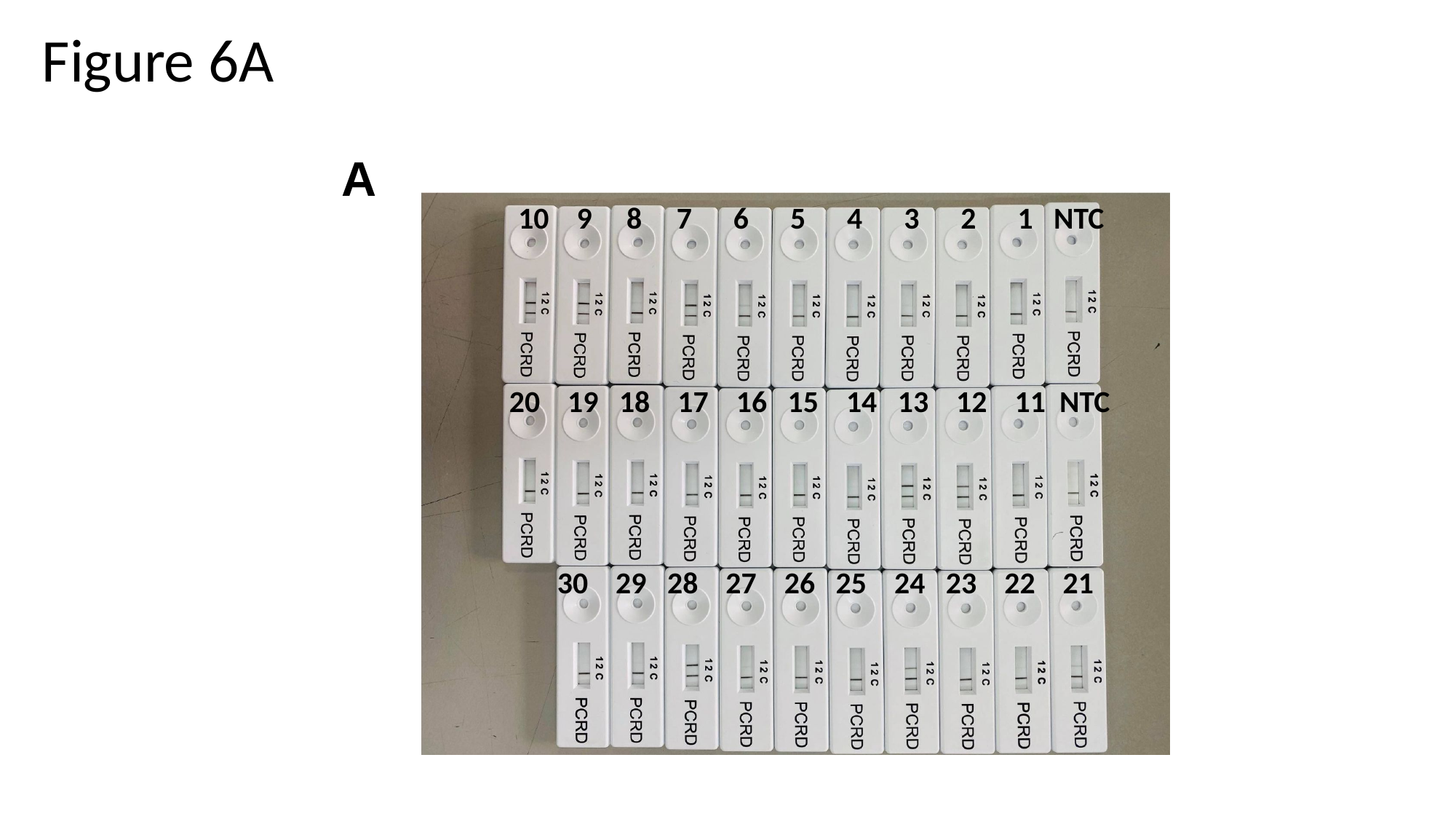

Figure 6A
A
10 9 8 7 6 5 4 3 2 1 NTC
20 19 18 17 16 15 14 13 12 11 NTC
30 29 28 27 26 25 24 23 22 21

## Slide 6
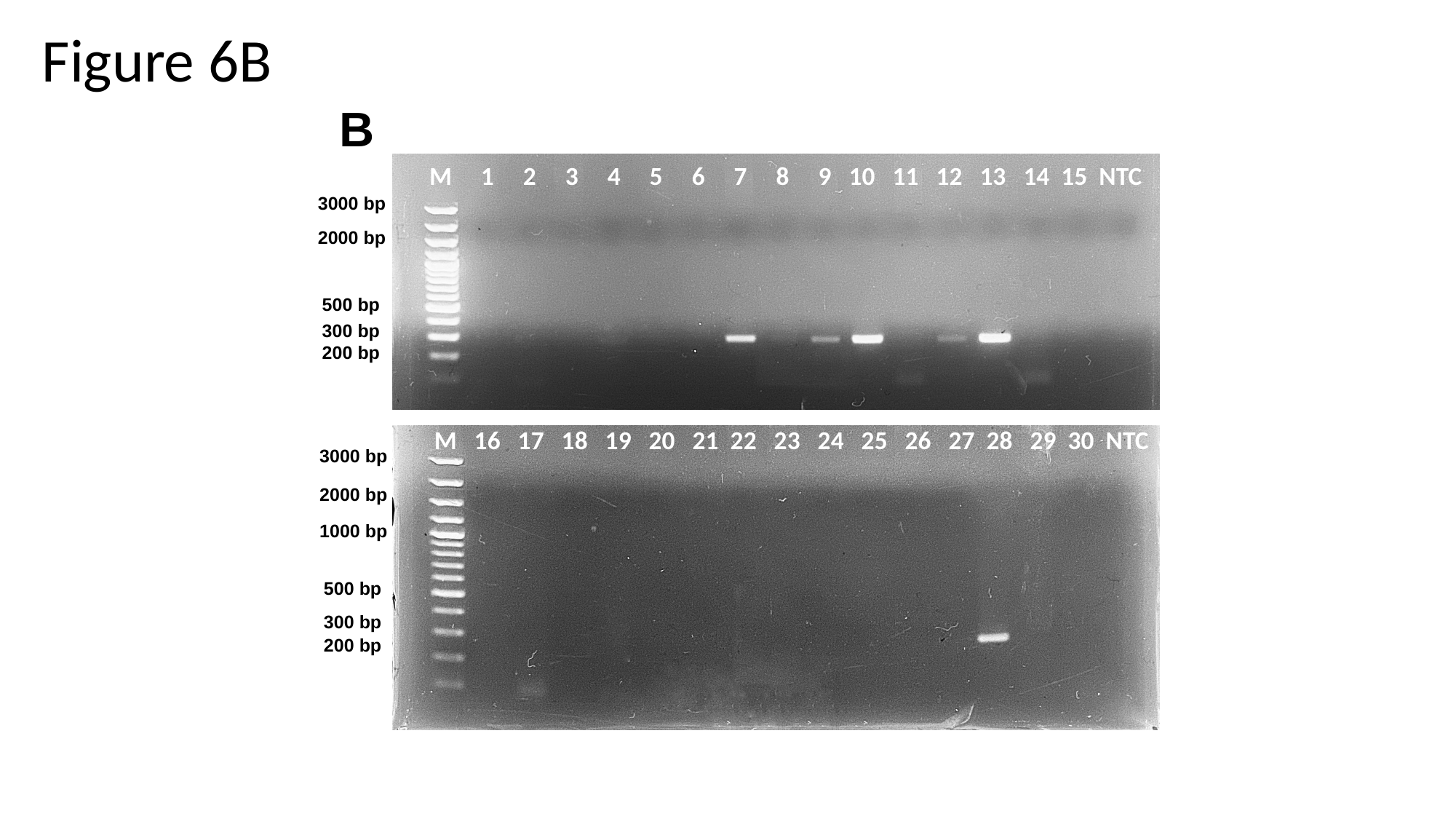

Figure 6B
B
M 1 2 3 4 5 6 7 8 9 10 11 12 13 14 15 NTC
3000 bp
2000 bp
500 bp
300 bp
200 bp
M 16 17 18 19 20 21 22 23 24 25 26 27 28 29 30 NTC
3000 bp
2000 bp
1000 bp
500 bp
300 bp
200 bp
