# Supplementary material for: Sensitive and rapid detection of Babesia species in dogs by recombinase polymerase amplification with lateral flow dipstick (RPA-LFD)
Source: Sci Rep. 2022 Nov 29;12:20560. doi: 10.1038/s41598-022-25165-7 (PMC9707278; doi:10.1038/s41598-022-25165-7)
Supplement: Supplementary file 2 — Supplementary Information 2. [file 41598_2022_25165_MOESM2_ESM.docx]

Supplemental Figure

**Sensitive and rapid detection of *Babesia* species in dogs by recombinase polymerase amplification with lateral flow dipstick (RPA-LFD)**

Warunya Onchan^1^, Onchira Ritbamrung^1^, Phanupong Changtor^1^, Waranee Pradit^2^, Siriwadee Chomdej^2^, Korakot Nganvongpanit^2,4^, Puntita Siengdee^2,5^, Urasri Suyasunanont^1^, Kittisak Buddhachat^1,3,*^

^1^Department of Biology, Faculty of Science, Naresuan University, Phitsanulok, Thailand, 65000

^2^Department of Biology, Faculty of Science, Chiang Mai University, Chiang Mai, Thailand, 50200

^3^Excellence Center in Veterinary Bioscience, Chiang Mai University, Chiang Mai, Thailand, 50200

^4^Department of Veterinary Biosciences and Public Health, Faculty of Veterinary Medicine, Chiang Mai University, Chiang Mai, Thailand, 50200

^5^Chulabhorn Graduate Institute, Program in Applied Biological Sciences, Chulabhorn Royal Academy, Kamphaeng Phet 6 Road, Laksi, Bangkok, Thailand, 10210

*Corresponding author

Kittisak Buddhachat, Ph.D.

Department of Biology, Faculty of Science, Naresuan University, Phitsanulok, Thailand, 65000

E-mail: [kittisakbu@nu.ac.th](mailto:kittisakbu@nu.ac.th), [k_buddhachat@yahoo.com](mailto:k_buddhachat@yahoo.com)

**Supplemental Figure 1** Multiple alignments of partial 18S rRNA sequences of different blood parasite species retrieved from GenBank, NCBI to determine the suitable primer for specific DNA amplification on *Babesia* spp. Two DNA binding sites were designed for primer sets as rpaBab264 (A) and rpaBab128 (B).

**Supplemental Figure 2** The comparison of different reaction volume of TwistAmp nfo kit at 50 μl and 25 μl to detect *Babesia* spp. from canine blood. NTC represents non template control.
